# Supramolecular Sensing of a Chemical Warfare Agents Simulant by Functionalized Carbon Nanoparticles

**DOI:** 10.3390/molecules25235731

**Published:** 2020-12-04

**Authors:** Nunzio Tuccitto, Luca Spitaleri, Giovanni Li Destri, Andrea Pappalardo, Antonino Gulino, Giuseppe Trusso Sfrazzetto

**Affiliations:** 1 Department of Chemical Sciences, University of Catania, Viale A. Doria 6, 95125 Catania, Italy; luca.spitaleri@phd.unict.it (L.S.); giolides@unict.it (G.L.D.); andrea.pappalardo@unict.it (A.P.); agulino@unict.it (A.G.); 2 Laboratory for Molecular Surfaces and Nanotechnology–CSGI, Viale A. Doria 6, 95125 Catania, Italy; 3 National Interuniversity Consortium for Materials Science and Technology (I.N.S.T.M.) Research Unit of Catania, Viale A. Doria 6, 95125 Catania, Italy

**Keywords:** carbon nanoparticles, chemical warfare agents, sensor, supramolecular recognition

## Abstract

Real-time sensing of chemical warfare agents by optical sensors is today a crucial target to prevent terroristic attacks by chemical weapons. Here the synthesis, characterization and detection properties of a new sensor, based on covalently functionalized carbon nanoparticles, are reported. This nanosensor exploits noncovalent interactions, in particular hydrogen bonds, to detect DMMP, a simulant of nerve agents. The nanostructure of the sensor combined with the supramolecular sensing approach leads to high binding constant affinity, high selectivity and the possibility to reuse the sensor.

## 1. Introduction

Chemical warfare agents (CWAs), organophosphorus compounds developed before the Second World War, are highly toxic compounds due to their ability to irreversibly inhibit the acetylcholine esterase enzyme [1].

**Scheme 1:**
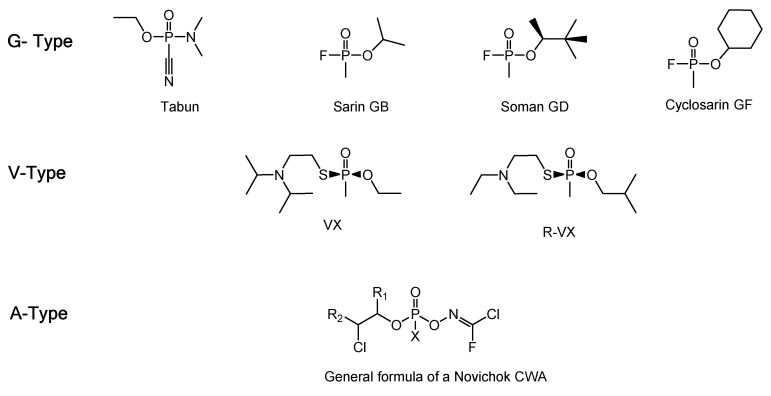
Structures and names of G-type, V-type and general formula of A-type chemical warfare agents (CWAs).

Today, CWAs are classified into three classes: G-type, V-type and A-type (also called Novichok) (Scheme 1).

Although CWAs are today prohibited in many countries, the recent attacks in the Middle East and in Europe highlight the importance to quickly detect, in real time, the presence of these agents [2,3].

Due to the toxicity levels of G- and V-type CWAs (LC50 values of Tabun and Sarin are 2 and 1.2 ppm, respectively, while Soman and VX have LC50 of 0.9 and 0.3 ppm, respectively) [4], the ability to detect low concentration values is of primary importance.

Research activity is usually performed by using simulants, less toxic compounds having geometries and sizes very similar to real CWAs [5]. In particular, the dimethyl methylphosphonate (DMMP) is today widely recognized as one of the best simulants for G-type CWAs.

Sensing of CWAs can be performed by using two different approaches: the “covalent approach” [6], based on a covalent reaction between the sensor and the analyte that leads to a change on a specific measurable property, or the “supramolecular approach” [7], in which the analyte is recognized by exploiting noncovalent interactions with a synthetic receptor. The covalent approach shows some limits: (i) low specificity, (ii) possibility of false-positive responses and (iii) impossibility to reuse the sensor. By contrast, the recent success of the supramolecular approach is due to the possibility to reuse the sensor, because of the formation of noncovalent interactions with the analyte [8,9,10,11,12,13,14,15,16], and the high efficiency (in terms of binding constant values) [17] and selectivity due to the possibility to recognize the analyte by multiple interactions (multitopic approach) [18,19].

Recently, we reported on the first metal-free fluorescent sensor, Naphthyl-Di-AE, able to selectively and reversibly detect DMMP via the multitopic approach by exploiting multiple hydrogen bonds [20]. Furthermore, very recently, we reported on the first nanosensor based on carbon nanoparticles (CNPs), able to detect very low concentration values of DMMP both in solution and in the gas phase [21]. The use of CNPs [22,23,24] for sensing purposes shows many interesting features, such as the high fluorescence quantum yield [25], water solubility, low toxicity [26], low-cost synthesis [27] and the possibility to functionalize their external shell [28].

**Scheme 2:**
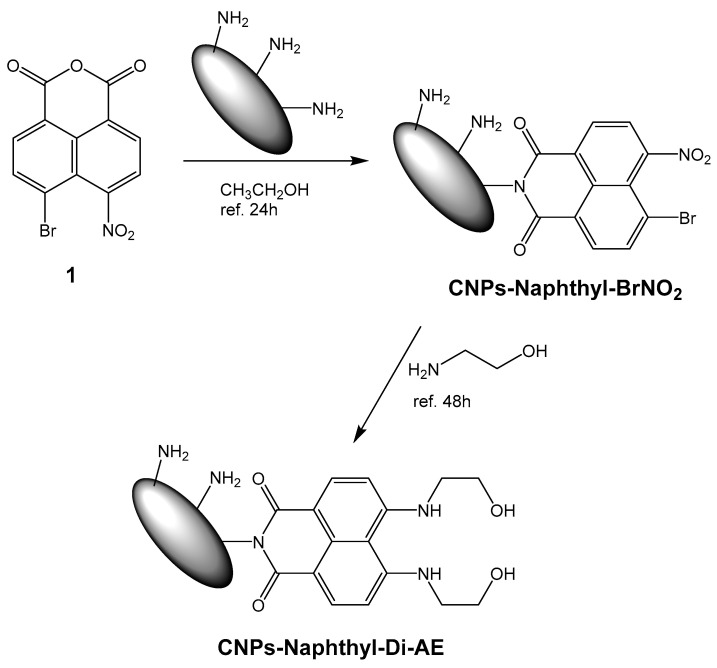
Synthesis of CNPs-Naphthyl-Di-AE.

Taking into account these considerations, here we present the implementation of the Naphthyl-Di-AE on the surface of carbon nanoparticles, leading to **CNPs-Naphthyl-Di-AE** (Scheme 2). The presence of a specific sensor for DMMP (Naphthyl-Di-AE) on the nanoparticle surface leads to the possibility to increase the selectivity towards the desired analyte target and to follow the recognition event by monitoring a specific change in the optical spectrum of the receptor.

In particular, the ethanolamine arms, responsible for hydrogen bond formation, are in the perfect geometry to recognize DMMP with both arms. With respect to our recent nanoparticle-based sensor, having the ethanolamine arms randomly bounded onto the nanoparticle surface [21], **CNPs-Naphthyl-Di-AE** show the recognition group (previously synthesized) having the optimal configuration to chelate and DMMP.

## 2. Results and Discussion

**CNPs-Naphthyl-BrNO_2_** were obtained by reacting the anhydride **1** [29] in refluxing ethanol with amino-terminal CNPs, and they were purified by centrifugation and filtration. Reaction of **CNPs-Naphthyl-BrNO_2_** with a large excess of ethanolamine in solvolysis leads to the **CNPs-Naphthyl-Di-AE**, which have been purified by centrifugation and dialysis. The ^1^H-NMR spectrum suggests the covalent functionalization with the ethanolamine groups, due to the presence of aromatic signals characteristic of the naphthalic core and, in the aliphatic region, of the typical pattern of ethanolamine (see Supplementary Material).

The electronic structure of the carbon nanoparticles (CNPs) functionalized with the naphthalic probe, **CNPs-Naphthyl-Di-AE**, was also investigated by X-ray photoelectron spectroscopy (XPS), thus providing information on the chemical environment. XPS also gave information on the covalent anchoring of the naphthalic sensor on the CNPs surface, appropriately functionalized with –NH_2_ groups. Finally, the surface elemental composition was estimated, once the relevant atomic sensitivity factors had been taken into account [30,31,32,33,34].

**Figure 1:**
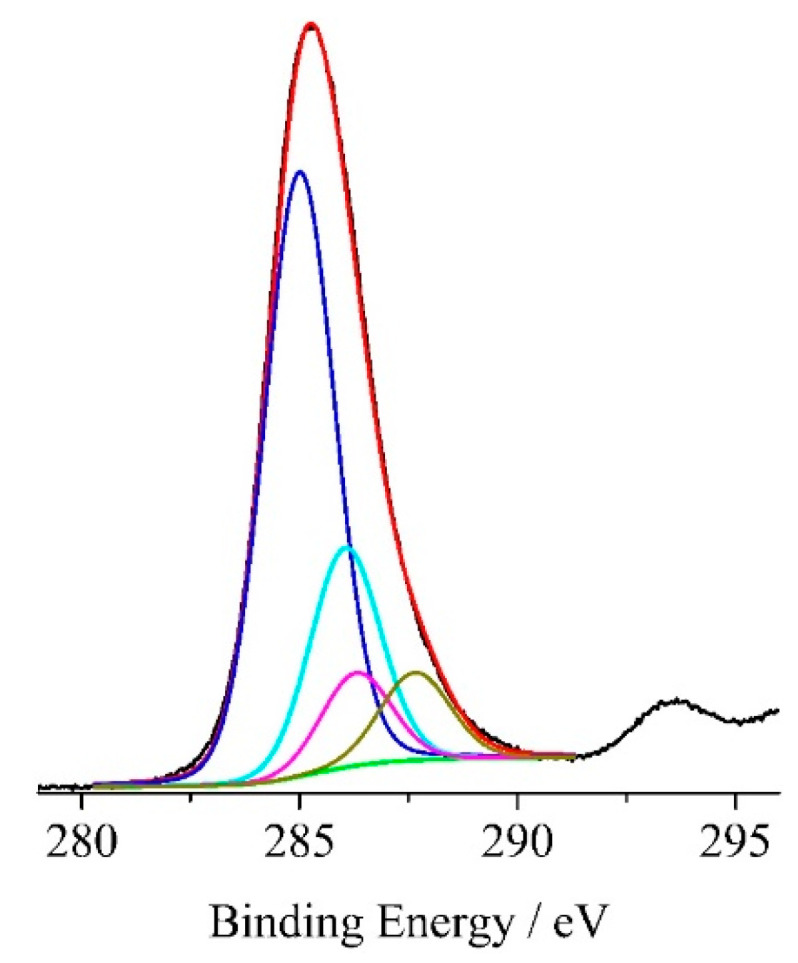
Al-Kα excited XPS of the CNPs–naphthalic anhydride sample in the C 1s binding energy region. The blue, cyan, magenta and dark yellow lines refer to the 285.0, 286.0, 286.4 and 287.7 components; the green line refers to the background, and the red line superimposed to the experimental black profile refers to the sum of all Gaussian components.

Figure 1 shows the high-resolution XPS spectrum of the **CNPs-Naphthyl-Di-AE** in the C 1s binding energy region. An accurate fitting of this spectrum revealed the presence of four components at 285.0, 286.0, 286.4 and 287.7 eV, respectively. The first component (285.0 eV) is due to both aliphatic and aromatic backbones [35,36]. The peaks at 286.0 eV and 286.4 eV are due to the C–N and C–OH groups, respectively [37,38]. The peak at 287.7 eV is assigned to the carbon of the amide group (–OC–NH–) [39], and the presence of this signal confirms the covalent functionalization of the CNPs with the naphthalic anhydride. Moreover, the intensity ratio between these three bands at 286.0, 286.4 and 287.7 eV is 5:2:2, and this result is in agreement with the chemical structure of the **CNPs-Naphthyl-Di-AE**, reported in Scheme 2. Finally, the peak at 293.2 eV is ascribed to π–π* shake-up satellites characteristic of the aromatic and conjugate systems.

**Figure 2:**
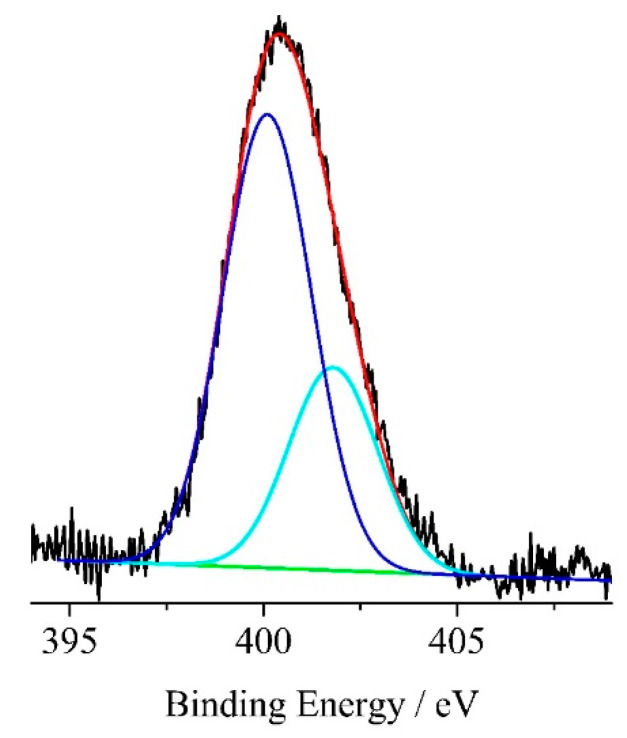
Al-Kα excited XPS of **CNPs-Naphthyl-Di-AE** in the N 1s binding energy region. The blue and cyan lines refer to the 400.1 and 401.7 eV components; the green line refers to the background, and the red line superimposed to the experimental black profile refers to the sum of all Gaussian components.

Figure 2 shows the XPS of **CNPs-Naphthyl-Di-AE** in the N 1s binding energy region. The N 1s spectral profile was fitted using two Gaussian components at 400.1 and 401.7 eV. The first component (400.1 eV) is due to the nitrogen of amine groups (–NH–) [39]. The peak at 401.7 eV is due to the nitrogen of the imide group (O=C–N–C=O) [40]. The presence of O=C–N–C=O group confirms the covalent functionalization of the CNPs with the naphthalic core. The intensity ratio between the two bands at 400.1/401.7 eV is 2:1, and this result is in agreement with the chemical structure of the naphthalic anhydride molecule.

**Figure 3:**
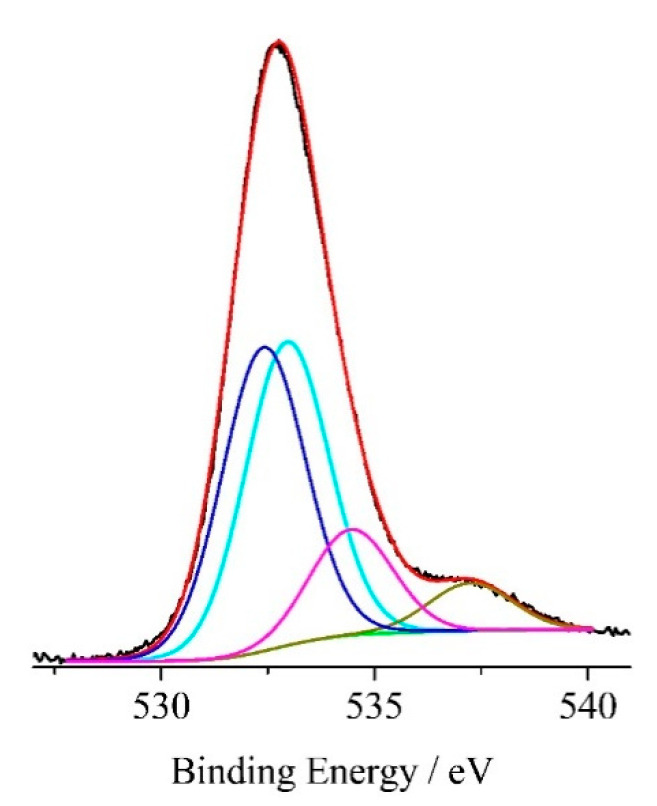
Al-Kα excited XPS of the **CNPs-Naphthyl-Di-AE** sample in the O 1s energy region. The black line refers to the experimental profile; the green line refers to the background; the Gaussian at 532.3 (blue line), 532.9 (cyan line), 534.4 (magenta line) and 537.3 eV (dark yellow line) represent the four O 1s components; the red line, superimposed to the experimental profile, refers to the sum of the Gaussian components.

Finally, Figure 3 shows the O 1s peak of the CNPs–naphthalic anhydride system. The O 1s spectral profile was fitted using four Gaussian components at 532.3, 532.9, 534.4 and 537.3 eV. The lower energy peak, located at 532.3 eV, is assigned to the –OH groups of both CNPs and naphthalic core [41]. The second peak at 532.9 eV is assigned to the imide (O=C–N–C=O) group [40,41] and further confirms the covalent functionalization of the CNPs with the naphthalic anhydride molecule. Once more, the intensity ratios of the first two peaks (2:2) presently observed are strongly in agreement with the expected intensity trend, on the basis of the structure of **CNPs-Naphthyl-Di-AE**. The higher energy peak located at 534.4 eV is attributed to the H_2_O molecules present in the air-exposed CNPs–naphthalic anhydride system. Besides, the 537.3 eV peak is attributed to the molecular O_2_ adsorbed on the surface of the air-exposed sample [41].

**Figure 4:**
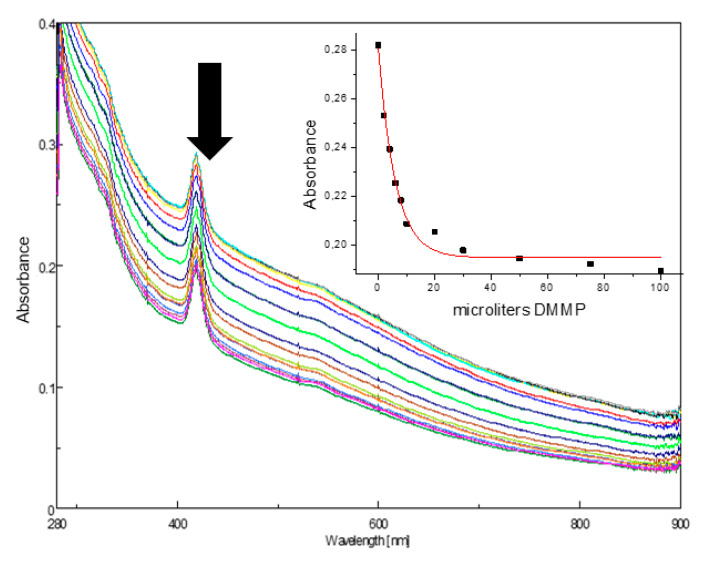
UV-Vis spectra of **CNPs-Naphthyl-Di-AE** (0.05 mg/mL in toluene) upon progressive addition of DMMP (0–100 μL of a 1 × 10^−4^ M solution in toluene), inset shows the calibration curve.

Sensing properties were evaluated by UV-Vis spectroscopy. A solution of 0.05 mg/mL of **CNPs-Naphthyl-Di-AE** in toluene shows a broad absorption band, characteristic of the carbon nanoparticles extending all over the visible and near UV region. In addition, the band relative to the Naphthyl-Di-AE sensor can be detected at 430 nm. This band was monitored during the sensing of DMMP. In particular, the progressive addition of DMMP (0–100 μL of a 1 × 10^−4^ M solution in toluene) leads to a decrease of the absorbance intensity (Figure 4), in agreement with the behavior observed by using the molecular sensor in solution [20]. The nonlinear curve fit of this data indicates a binding constant affinity of log 5.55 ± 0.30, more than one order of magnitude higher with respect to that of the Naphthyl-Di-AE in solution (log 4.02), thus demonstrating the higher performance of the nanosensor with respect to the molecular sensor. Notably, the calculated detection limit is 0.16 ppm (see Section 3), lower than the LC50 values of the most common CWAs used (fluorescence measurements are precluded due to the stronger emission of the carbon nanoparticle core that overlaps the emission of the naphthalic core).

**Figure 5:**
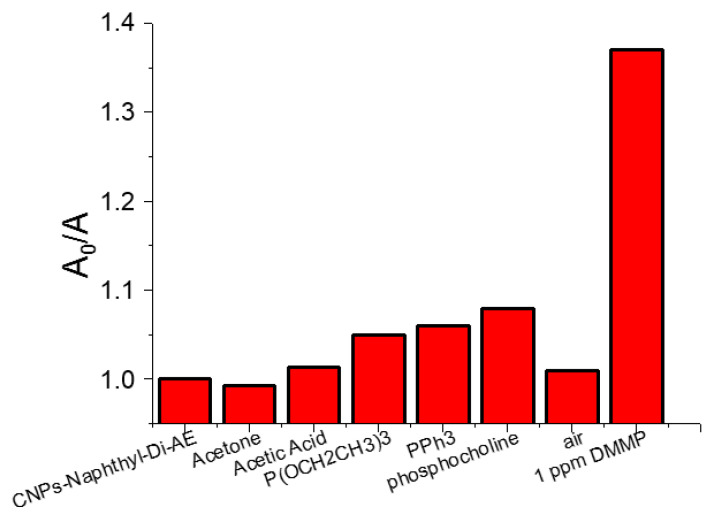
Normalized UV-Vis responses of **CNPs-Naphthyl-Di-AE** (0.05 mg/mL in toluene) to various competitive guests: 50 ppm of acetone, acetic acid, triethylphosphite (P(OCH_2_CH_3_)_3_), triphenylphosphine (PPh_3_) and phosphocholine; air (bubbled for 30 min); and DMMP (1 ppm). Bars represent the initial over the final absorbance values at 430 nm.

Selectivity is a crucial parameter for a sensor. For this reason, we tested the UV-Vis response of **CNPs-Naphthyl-Di-AE** to different organic analytes, in order to test the efficiency of the multitopic approach. Figure 5 shows the normalized absorbance values of **CNPs-Naphthyl-Di-AE** observed upon the addition of 50 ppm of different analytes. We note that carbonyl compounds do not interact with our sensor. Organic phosphorous compounds, such as triethylphosphite, triphenylphosphine and phosphocholine cause a slight increase of the optical response. The presence of air (containing 24,000 ppm of water, 400 ppm CO_2_, 5 ppm NO and 10 ppm CO), bubbled for 30 min, does not interfere with the nanosensor. On the contrary, the addition of 1 ppm of DMMP leads to a strong optical response.

## 3. Materials and Methods

### 3.1. General Experimental Methods

The NMR experiments were carried out at 27 °C on a Varian UNITY Inova 500 MHz spectrometer (^1^H at 499.88 MHz, ^13^C-NMR at 125.7 MHz, Varian-Agilent, Santa Clara, CA, USA) equipped with a pulse-field gradient module (Z axis) and a tunable 5 mm Varian inverse detection probe (ID-PFG). UV-Vis measurements were carried out using a JASCO V-630 spectrophotometer (Mettler Toledo, Novate Milanese, Italy) at room temperature. All chemicals were reagent grade (Signa Aldrich, Buchs, Switzerland) and used without further purification. X-ray photoelectron spectra (XPS) were measured at a 45° take-off angle relative to the surface normal with a PHI 5600 Multi Technique System (Physical Electronics GmbH, Feldkirchen, Germany, base pressure of the main chamber 3 × 10^−8^ Pa) [35,36]. Samples were excited with the Al-Kα X-ray radiation using a pass energy of 5.85 eV. Structures due to the Kα satellite radiations were subtracted from the spectra prior to data processing. Spectra calibration was achieved by fixing the Ag 3d5/2 peak of a clean sample at 368.3 eV; this method turned the C 1s main peak at 285.0 eV [35,36,42]. The instrumental energy resolution was ≤ 0.5 eV. The XPS peak intensities were obtained after Shirley background removal [35,36]. The atomic concentration analysis was performed by taking into account the relevant atomic sensitivity factors. The fittings of the C 1s, N 1s and O 1s XP spectra were carried out using Gaussian envelopes after subtraction of the background until there was the highest possible correlation between the experimental spectrum and the theoretical profile. The residual or agreement factor *R*, defined by *R* = [Σ(F_obs_ − F_calc_)^2^/Σ(F_obs_)^2^]^1/2^, after minimization of the function Σ(F_obs_ − F_calc_)^2^, converged to the value of 0.03. The fitting was performed using the XPSPEAK4.1 software (free, fully featured, software for the analysis of XPS spectra written by Raymund Kwok). Samples for XPS measurement were deposited on silicon substrates.

### 3.2. Synthesis of CNPs-Naphthyl-BrNO_2_

One hundred milligrams of amino-terminal CNPs [43] was dissolved in 15 mL of absolute ethanol, and 70 mg (0.217 mmol) of **1** was added. The mixture was stirred at reflux for 24 h under nitrogen atmosphere. Solvent was removed by rotavapor, and the **CNPs-Naphthyl-BrNO_2_** were purified by centrifugation and filtration in hot ethanol.

### 3.3. Synthesis of CNPs-Naphthyl-Di-AE

Twenty milligrams of **CNPs-Naphthyl-BrNO_2_** was dissolved in 10 mL of ethanolamine, and the mixture was refluxed under nitrogen atmosphere for 48 h. The excess of ethanolamine was removed under reduced pressure, and the **CNPs-Naphthyl-Di-AE** were purified by centrifugation and dialysis.

### 3.4. Procedure for UV-Vis Titrations

From a guest stock solution (1.0 × 10^−4^ M) in toluene, different volumes (0, 2, 4, 6, 8, 10, 20, 30, 50, 75, 100 μL) were added to the host (0.05 mg/mL in toluene), and the UV-Vis spectra were recorded at 25 °C. The apparent binding affinities of **CNPs-Naphthyl-Di-AE** with DMMP were estimated by using HypSpec (version 1.1.33) [44,45,46], a software designed to extract equilibrium constants from potentiometric and/or spectrophotometric titration data. HypSpec starts with an assumed complex formation scheme and uses a least-squares approach to derive the spectra of the complexes and the stability constants. χ^2^ test (chi-square) was applied, where the residuals followed a normal distribution (for a distribution approximately normal, the χ^2^ test value is around 12 or less). In all of the cases, χ^2^ ≤ 10 was found, as obtained by 3 independent measurement sets. Limit of detection was calculated by the method of the calibration curve using the formula DL = 3s/K, where s is the standard deviation of the blank and K is the slope of the calibration curve.

## 4. Conclusions

In the present study, we report the covalent functionalization of carbon nanoparticles by a selective sensor of Chemical Warfare Agents. The presence of a specific sensor for DMMP (Naphthyl-Di-AE) on the carbon nanoparticle surface leads to the possibility to increase the selectivity towards the given analyte target and to follow the recognition event by monitoring a specific change of the optic spectrum of the receptor. The new nanosensor shows high binding affinity towards DMMP, high selectivity and a sub-ppm detection limit. The possibility to tune the molecular structure of the external shell of the carbon nanoparticles leads to a wide range of applications, including sensing applications. Studies are ongoing to obtain functionalized fluorescent carbon nanoparticles suitable for real prototypes to be commonly used.

## Supplementary Materials

The following are available online. Figure S1: 1H-NMR spectrum of **CNPs-Naphthyl-Di-AE** in DMSO-*d_6_*; Figure S2: UV-Vis titration between **CNPs-Naphthyl-Di-AE** and DMMP.
