# Supplementary material for: Supramolecular Sensing of a Chemical Warfare Agents Simulant by Functionalized Carbon Nanoparticles
Source: Molecules. 2020 Dec 4;25(23):5731. doi: 10.3390/molecules25235731 (PMC7730470; doi:10.3390/molecules25235731)
Supplement: Supplementary file 1 [file molecules-25-05731-s001.pdf]

Supramolecular Sensing of a Chemical Warfare Agents Simulant by Functionalized Carbon Nanoparticles

Nunzio Tuccitto*, Luca Spitaleri, Giovanni Li Destri, Andrea Pappalardo, Antonino Gulino and Giuseppe Trusso Sfrazzetto*

Supplementary Material

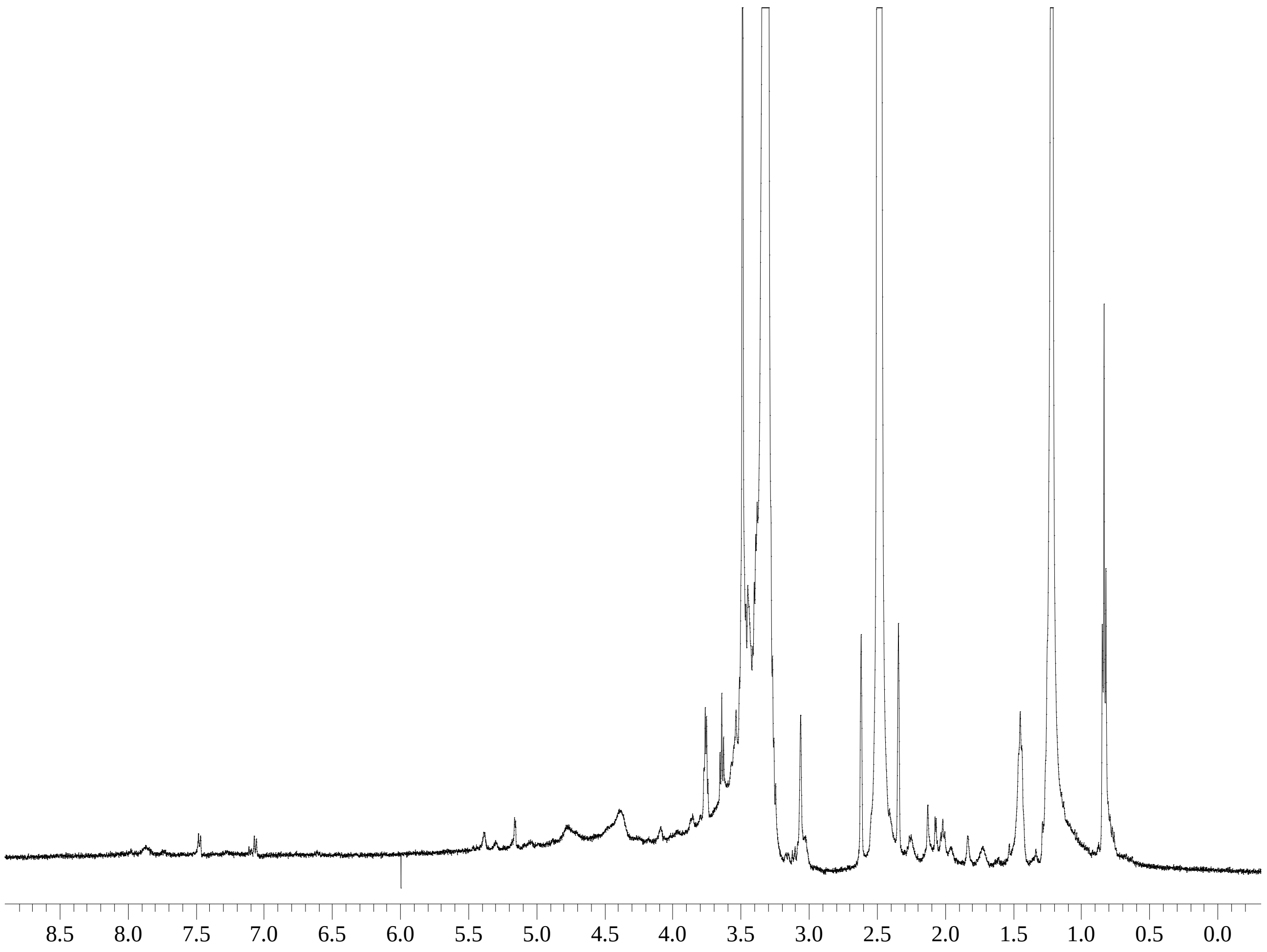

Figure S1. ^{1}H NMR spectrum of **CNPs-Naphthyl-Di-AE** in DMSO-d_6

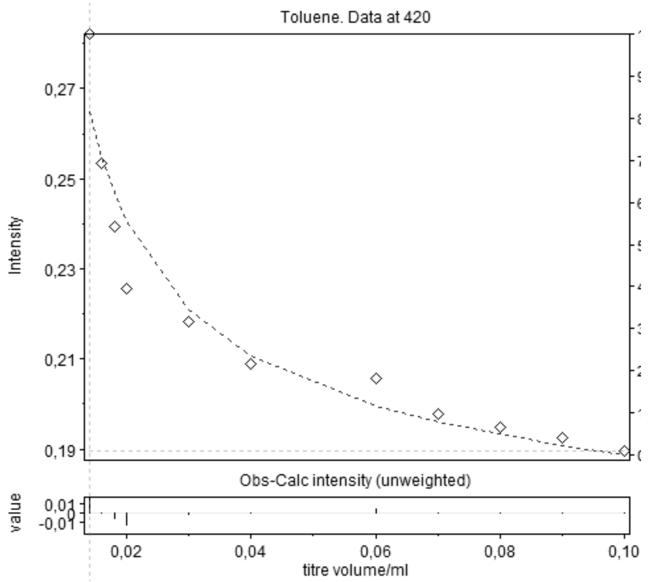

HypSpec output file

```
Converged in 1 iterations with sigma = 0,013543

                     standard
Log beta   value     deviation
AB         5.5465    0.2979
```

Figure S2. UV-Vis titration between **CNPs-Naphthyl-Di-AE** and DMMP
